# Novel STAT binding elements mediate IL-6 regulation of MMP-1 and MMP-3

**DOI:** 10.1038/s41598-017-08581-y

**Published:** 2017-08-17

**Authors:** Samuel J. Cutler, James D. Doecke, Ibtisam Ghazawi, Jinbo Yang, Lyn R. Griffiths, Kevin J. Spring, Stephen J. Ralph, Albert S. Mellick

**Affiliations:** 1 School of Medical Science, Griffith Institute for Health and Medical Research, Griffith University, Parklands Drive, Southport, 4215 QLD Australia; 2 Department of Molecular Genetics, Lerner Research Institute, 9500 Euclid Avenue, Cleveland, Ohio 44195 USA; 3 Institute for Health & Biomedical Innovation, Queensland University of Technology, 60 Musk Avenue, Kelvin Grove, QLD 4059 Australia; 4 School of Medicine, Western Sydney University, Locked Bag 1797, Penrith, NSW 2751 Australia; 5 Ingham Institute for Applied Medical Research, South Western Sydney Clinical School UNSW & CONCERT Translational Cancer Research Centre, 1 Campbell Street, Liverpool, NSW 2170 Australia

## Abstract

Dynamic remodelling of the extracellular matrix (ECM) is a key feature of cancer progression. Enzymes that modify the ECM, such as matrix metalloproteinases (MMPs), have long been recognised as important targets of anticancer therapy. Inflammatory cytokines are known to play a key role in regulating protease expression in cancer. Here we describe the identification of gamma-activated site (GA﻿S)-like, signal transducer and activator of transcription (STAT) binding elements (SBEs) within the proximal promoters of the *MMP-1* and *MMP-3* genes, which in association with AP-1 components (c-Fos or Jun), bind STAT-1 in a *homodimer* like complex (HDLC). We further demonstrate that MMP expression and binding of this complex to SBEs can either be enhanced by interleukin (IL)-6, or reduced by interferon gamma (IFN-γ), and that IL-6 regulation of MMPs is not STAT-3 dependent. Collectively, this data adds to existing understanding of the mechanism underlying cytokine regulation of MMP expression via STAT-1, and increases our understanding of the links between inflammation and malignancy in colon cancer.

## Introduction

The cancer microenvironment is made up of many host derived non-tumor cells that play an important role in driving cancer progression by mediating processes as diverse as angiogenesis^1, 2^, fibrosis^3^, and metastatic spread^4^. They do this by producing growth factors and cytokines that drive malignant cancer cell gene expression changes^5^; principally via phosphorylation (activation) of a family of cytoplasmic effectors, the signal transducers and activators of transcription (STATs)^6, 7^. Upon activation STATs mobilise to the nucleus and recognise defined sequences within the promoters of target genes, referred to as STAT binding elements (SBEs).

Historically, SBEs were determined by identifying conserved sequences in the promoter regions of interferon (IFN) stimulated genes (ISGs)^8, 9^. DNA binding sites for STATs in the promoters of genes induced by type I IFN^10^, type II IFN and interleukin (IL)-6 were further confirmed by electrophoretic mobility shift assays (EMSA)^11^ and mutational analysis^12^. In this way, the type I IFN-activated complex (ISGF3) was found to recognise a direct repeat consensus sequence GAAANNGAAANN, referred to as the IFN-stimulated response element (ISRE), and the type II IFN-γ activated complex (aka γ-activated factor) was shown to recognise the sequence TT(C/A)CNN(G/T)AA, referred to as the IFN-γ-activated sequence (GAS). In addition, both STAT-3 homodimers and STAT-1/3 heterodimers are capable of binding to GAS-like sequences, but with subtle differences in affinity^12^. As a consequence, IL-6 treatment of a responsive tissue will lead to phosphorylation of either STAT-1 and/or STAT-3, which in turn bind to SBEs in either a homodimer, or heterodimeric formation^13^. While treatment with IFN-γ leads to activation of GAS-like SBEs in ISGs, through the activation and binding of homodimers containing phosphorylated STAT-1 (P-STAT-1)^14, 15^. Notably, unphosphorylated STATs (U-STATs) are also known to play various roles in regulating gene expression, although the mechanism for this remains relatively undefined^16–18^.

The Matrix Metalloproteinase (MMP) family have a diverse range of substrate specificities related to remodelling of the extracellular matrix (ECM). Aberrant MMP activity has been implicated in a range of malignancies, such as those of the colon and breast^19, 20^, where they act to promote malignancy by degrading basement membranes, and by activating ECM-bound growth factors and cytokines^21^. MMP family members have been identified as targets of inflammatory cytokinemediated gene regulation via STAT signaling^22, 23^. These include, *Collagenase I* (*MMP-1*), which is proposed to be regulated by Oncostatin M (OSM) via STAT-3 binding to an SBE located in a region of the human gene promoter proximal to the start site of transcription^24, 25^, as well as *MMP-3* (Stromelysin), which appears to be regulated by IL-6 via a distal SBE^26, 27^. *Gelatinase A* (*MMP-*2) and *Gelatinase B* (*MMP-9*) gene activity are also thought to be regulated by cytokines via SBEs^28^.

In this study, we present data showing that IL-6 regulates MMP expression via proximal GAS-like SBEs, and that IL-6 treatment leads to binding of a novel complex, which contains both STAT-1 and components of activated protein (AP)-1, but not STAT-3. Notably, ablation or inhibition of STAT-1, not STAT-3 was found to inhibit IL-6-mediated induction of MMPs. This work complements previous studies that show a role for STAT-3 in maximal MMP induction via IL-6^29, 30^, by suggesting that STAT-3 functionality is not a precondition for IL-6-mediated MMP expression. This finding also provides a novel insight into inflammatory cytokine signaling and represents an important consideration for the development of anti-cancer drugs targeting STAT-mediated cytokine signaling^31^.

## Results

### Protease and inflammatory cytokine signaling in colon cancer

**Figure 1:**
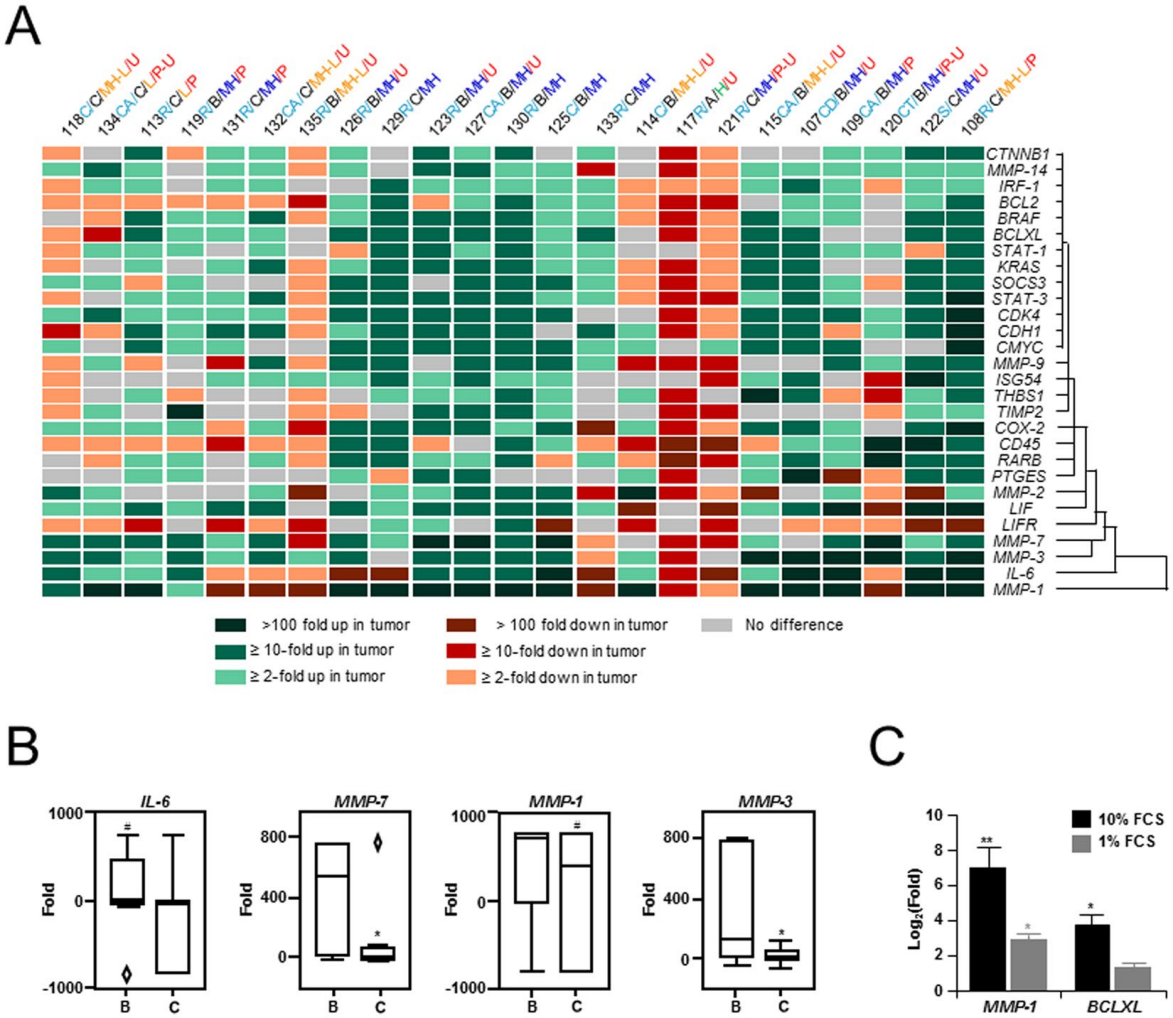
Comparative expression analysis. (**A**) Heat map showing relative fold differences in gene expression between tumor-tissue and patient matched normal mucosa. Clustering was based on nearest neighbor hierarchy analysis. Where expression was below the level of sensitivity for detection in either the normal mucosa or tumor, values were assigned numerically reflecting the upper and lower bounds of the data set (no expression in control, 800; no expression in tumor, −800). Data was analyzed by Spearman’s ρ correlation analysis (α = 0.05). Abbreviations for anatomical location: Cecum [C]; Colon Ascending [CA]; Colon Transverse [CT]; Colon Descending [CD]; and Sigmoid Colon [S]; for Dukes’ staging [B & C]; for differentiation: Low [L]; Medium High to Low [MH-L]; Medium High [MH]; and High [H]; for ulceration: Ulcerous [U]; Polypous [P]; Mixed Polypous-Ulcerous [P-U]. (**B**) Results of Q-PCR analysis showing differences in mRNA levels f﻿or *MMP-1*, *MMP-3*, *MMP-7 *and *IL-6* between Dukes’ B and Dukes’ C tumors. Data is represented as box plots with median (thick line), first and third quartiles (boxed), upper and lower values (whiskers) and outliers (◊), and was analyzed by Mann Whitney U (α = 0.05, *P < 0.05). (**C**) Results of Q-PCR analysis following IL-6 treatment of SW480 colorectal cancer cells, showing significant up-regulation of *MMP-1* mRNA. Notably, induction was enhanced under high (10%) serum conditions (*Left*), compared with low serum (1%) (*Right*). Similar results were obtained in other cell lines, including LS174T (*not shown*). Also shown, the effects of IL-6 on the known IL-6 target gene, *BCLXL*. Similar results were obtained for *BCL-2* and *STAT-3 (not shown)*. Each experiment was repeated showing similar results, and data represented as mean Log_2_(Fold) ± SEM, and was analyzed by Independent *t* test (α = 0.05, **P < 0.01).

Patient colon tumor tissue and matched normal mucosa were collected and analyzed by specific expression analysis (Fig. 1A and Supplementary Table S1). Genes were chosen based on previous association with colorectal cancer, and included inflammatory factors as well as oncogenes. While mRNA from oncogenes with known regulatory links such as c-MYC/STAT-3 and c-MYC/CDK4 showed co-regulation of expression, analysis also revealed significant correlation in the mRNA levels of *IL-6* and *MMPs*: *MMP-1*: P = 0.02 & *MMP-3*: P < 0.01, with generally higher levels in tumor tissue compared with normal mucosa (Supplementary Fig. S1A). Notably, specific comparison of gene expression revealed reduced levels of *MMP* and *IL-6* mRNA in Dukes’ C compared with Dukes’ B tumors, suggesting that co-regulation was also linked to clinical pathology (P < 0.05) (Fig. 1B)^32^. To examine this link further, levels of known downstream targets of IL-6 such as *BCL-2*, *STAT-3* and *BCLXL* ^33^, as well as *MMP-1* and *MMP-3*, were then measured following treatment of colon cancer cell lines with IL-6. Notably, *MMP-1* (~250-fold, P < 0.01) and IL-6 responsive *BCLXL* mRNAs were greatly increased after treatment with IL-6 (18 h) in SW480 colon cancer cells under conditions of high (10%) serum (Fig. 1C). In the presence of low (1%) serum, *MMP-1* and *MMP-3* mRNA expression remained inducible by IL-6, although the degree of induction was markedly reduced (~7-fold, P < 0.05). Similar results were obtained for *MMP-3*, and the IL-6 target gene, *BCL-2* (*not shown*). Furthermore, both LS174T and SW480 colon cancer cell lines were confirmed to be responsive to IL-6: evidenced by the induction of IL-6 responsive genes, and expression of IL-6 receptors (gp130 & IL-6R). Notably, although all cell lines examined constitutively expressed LIF, as well as mRNA for both IL-6 receptors, *IL-6* expression was found to be below the level of detection (Supplementary Fig. S1B). In addition, the HT29 cell line, which was the only cell line shown to express mRNA for the LIF receptor (LIFR), also showed an increase in *MMP-1* levels after LIF treatment (5.6-fold, P < 0.02). The presence of a previously reported GAS-like SBE in the proximal promoter of *MMP-1* (Supplementary Tables S2A)^25, 34–43^ also suggested that *MMP-1* might respond positively to IFN-γ treatment. However, although all the tested colon cancer cell lines were responsive to IFN-γ, as revealed by induction of ISGs^14, 15^ such as *STAT-1*, *STAT-3*, *interferon responsive factor (IRF-1)*, and *BCLXL*, unexpectedly *MMP-1* mRNA levels were reduced following IFN-γ treatment (18 h) (Supplementary Fig. S1C).

### Characterization of a non-canonical GAS-like SBE in the *MMP-1* promoter

**Figure 2:**
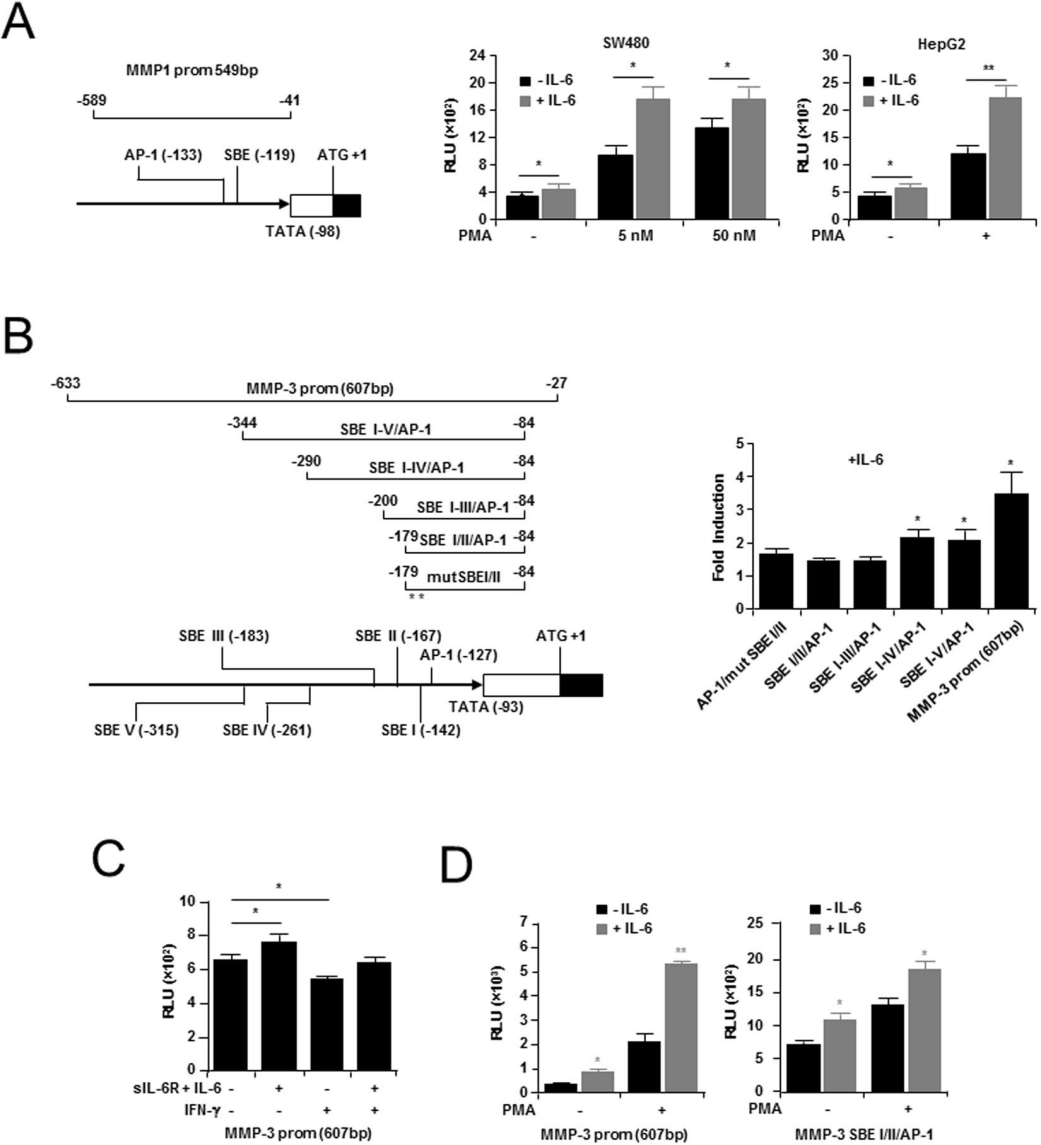
Reporter analysis of human *MMP* SBEs. (**A**) Schematic representation of the SBE/AP-1 region from the *MMP-1* promoter inserted into the pGL3-basic luciferase reporter vector (*Left*). Also shown, results of reporter activity in SW480 cells, transfected with the *MMP-1* promoter (549 bp) reporter following treatment with either Interleukin (IL)-6 alone, or IL-6 plus phorbol myristyl acetate (PMA); as well as results of reporter activation in cytokine responsive liver cancer (HepG2) cells, treated with IL-6 alone or IL-6 plus PMA (5 mM). Data is represented as mean relative luminescence Units (RLU) ±SEM. (**B**) *Left*, Schematic of human *MMP-3* promoter constructs used for dual reporter analysis, including the positions of SBEs and AP-1 binding elements. ‘*’Indicates mutations created in the SBEI and SBEII sites. *Right*, IL-6 induced promoter activity in HepG2 cells for each construct. Data is represented as mean fold induction ± SEM. (**C**) SW480 and HepG2 cells transfected with the proximal *MMP-3* promoter were treated with: (i) IL-6 and soluble IL-6 receptor (IL-6R) (both 10 ng/ml, 18 h); (ii) IFN-γ (1000 U/ml, 18 h) alone; or (iii) with IFN-γ, 2 h before adding IL-6 and sIL-6R. Data is represented as mean RLU ± SEM. (**D**) Luciferase activity in HepG2 cells produced from the full length 607 bp proximal region (*Left*), and the shortest SBE I/II/AP-1 (*Right*) *MMP-3* gene promoter construct. Shown, the cumulative effect of PMA (5 nM) plus IL-6 (10 ng/ml), versus IL-6 alone. Data is represented as mean RLU ± SEM. For (**A**–**D**), experiments were repeated showing similar results and data was analyzed by Independent *t* test (α = 0.05; *P < 0.05, **P < 0.01).

To further characterize the non-canonical GAS-like SBE element the *MMP-1* promoter sequences from human, mouse, dog, chimpanzee and rat were examined. Alignment of these sequences showed that the GAS-like SBE present in the proximal human *MMP-1* promoter^25^, as well as the nearby AP-1 binding element^44^ were highly conserved (Supplementary Fig. S2). We next cloned a 549 bp region of the *MMP-1* promoter containing this GAS-like SBE, and subjected the construct to dual reporter luciferase analysis (Fig. 2A)^45^. The results of this analysis confirmed that the proximal *MMP-1* promoter containing the GAS-like SBE was inducible by IL-6 in SW480 (1.2-fold) colon cancer cells, under conditions of low (1%) serum. This correlated with results seen in the cytokine responsive HepG2 liver cancer cell line, which showed a 3-fold induction. Next, to replicate the effects of serum response to *MMP* induction with IL-6 (Figs 1C and S3A) the activator of AP-1, phorbol myristyl acetate (PMA) was also added^46^. In this instance, addition of PMA provided significantly greater *MMP-1* promoter activity than addition of IL-6 alone (Fig. 2A); a result that is consistent with PMA promoting the response of *MMP-1* to IL-6 by co-stimulating AP-1 transcriptional activity.

### Characterization of novel SBEs in the human *MMP-3* proximal promoter

Given the presence of an IL-6/cytokine responsive SBE in *MMP-1* (Fig. 2A)^25^, the proximal promoter of *MMP-3* was examined to determine if similar elements could also explain *MMP-3* induction by IL-6. In this way, a spatially conserved region near the transcription start site was found to contain a putative GAS-like SBE (SBE I) in close proximity to a canonical AP-1 binding site. In addition, four other SBE-like sequences (SBEII-V) were identified upstream of the SBE/AP-1 motif in the *MMP-3* promoter (Supplementary Fig. S2 and Supplementary Tables S2B,C). Notably, no SBEII-V matching sequences were identified in the *MMP-1* proximal promoter. Next, to determine whether these novel non-canonical *MMP-3* SBEs were responsive to IL-6 a 607 bp fragment, as well as successively smaller segments, were subjected to luciferase reporter analysis (Fig. 2B). Notably, a significant response to IL-6 treatment was identified in HepG2 cells transfected with the 607 bp region of the *MMP-3* promoter that contained both SBEI/AP-1 and SBEII-V elements (3.4-fold). Mutation of both SBE I/II sites in the shortest fragment did not change the magnitude of induction by IL-6. However, extending the promoter length to include the other SBE sites [particularly SBE IV (−261)] showed a cumulative and positive effect on IL-6-induced luciferase activity. Activation by IL-6 of the full length construct containing all proximal SBEs was confirmed in SW480 cells, in the presence and absence of sIL-6R: added to ensure maximal induction (Fig. 2C). Significantly, IFN-γ treatment (18 h) reduced *MMP-3* 607 bp promoter activity in SW480 cells, while pre-treating cells for 2 h with IFN-γ before adding IL-6 then inhibited IL-6-induced promoter activation (Fig. 2C). Consistent with the supportive role played by AP-1 in IL-6-mediated *MMP-1* expression, PMA also greatly enhanced the IL-6-mediated activation of both the 607 bp (6.4-fold), and the shorter SBE I/II/AP-1 (1.8-fold) *MMP-3* promoter fragments (Fig. 2D). Notably, addition of sIL-6R did not significantly alter IL-6 induction of *MMP-3* (607 bp) promoter (Supplementary Fig. S3B).

### Constitutive and enhanced binding of a novel complex containing STAT-1 and AP-1 components to the *MMP-3* proximal promoter

**Figure 3:**
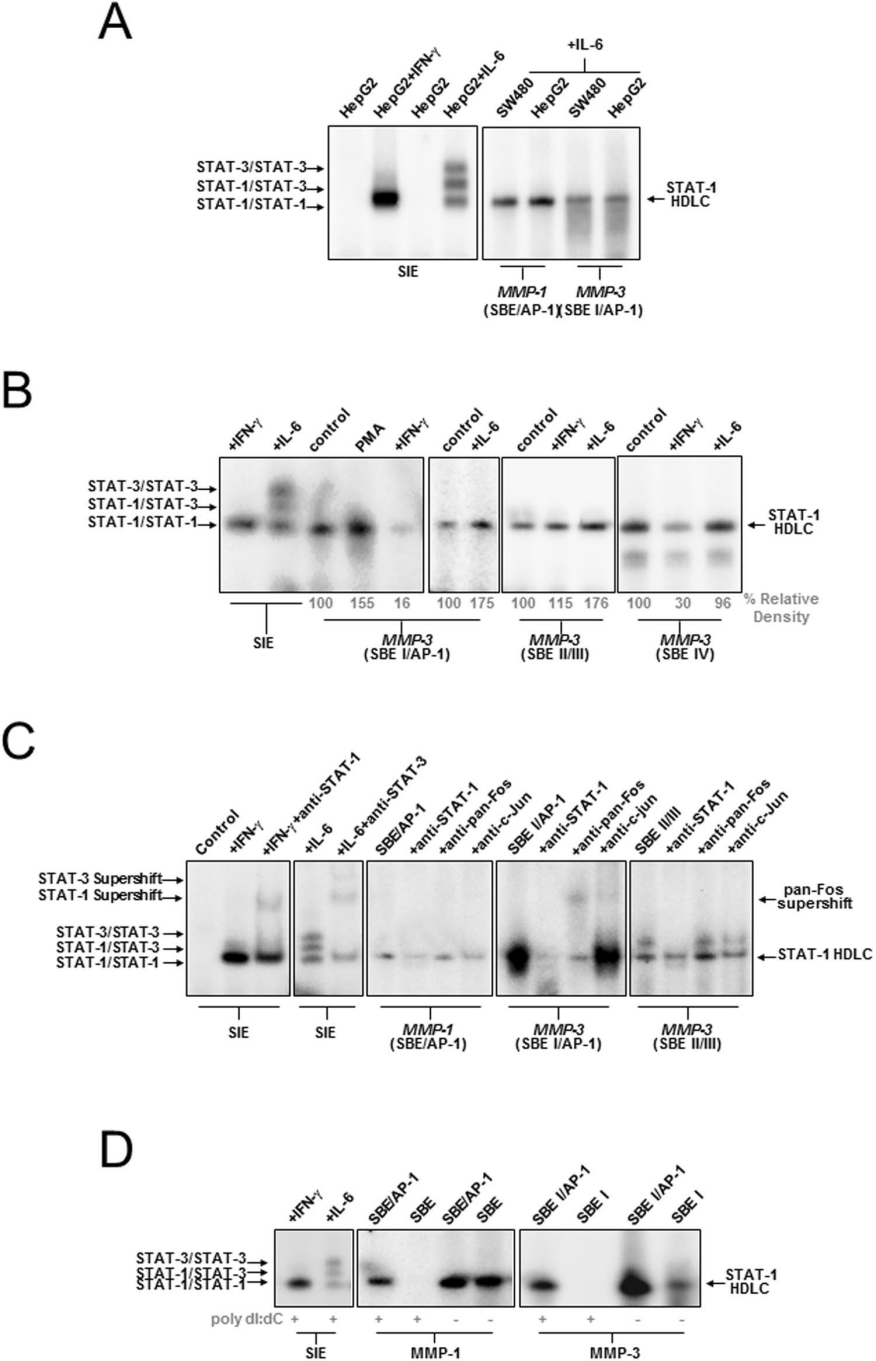
Identification of a HDLC binding non-canonical SBEs in *MMP-1 and MMP-3* promoters. (**A**) Extracts from SW480 or HepG2 cells treated with IL-6 (50 ng/ml, 5 min) or IFN-γ (1000 U/ml, 1 h) were probed with the *sis*-inducible element (SIE) oligonucleotide. Arrows indicate the STAT-3 homodimer, the STAT-1/STAT-3 heterodimer and the STAT-1 homodimer. Also shown is binding of a novel STAT-1 HDLC constitutively to the *MMP-1* SBE/AP-1 and *MMP-3* SBE I/AP-1 probes. (**B**) EMSA showing a similar STAT-1 HDLC constitutively binding to the *MMP-3* SBE II/III and *MMP-3* SBE IV probes using extracts from HepG2 cells treated with PMA (100 nM, 90 min), IL-6 (50 ng/ml, 5 min) and IFN-γ (1000 U/ml, 60 min). (**C**) EMSA in the presence of anti-STAT-1, anti-pan-Fos and anti-c-Jun antibodies, respectively, as indicated. (**D**) EMSA of *MMP1* (SBE) and (SBE/AP-1), *MMP-3* (SBEI) and (SBE I/AP-1) probes using HepG2 cell extracts in the presence and absence of 1 μg poly-dI:dC showing dependence of STAT-1 HDLC binding on the AP-1 site. For (**A–D**), experiments were repeated showing similar results.

To examine the mechanism for IL-6 activation of *MMP-1* and *MMP-3*, oligonucleotides containing the AP-1-proximal SBE from the *MMP-1* and *MMP-3* promoters (SBE/AP-1 & SBE I/AP-1, respectively) were subjected to EMSA to investigate direct DNA binding. As controls for STAT-binding, extracts from IFN-γ and IL-6-treated cells were incubated with oligonucleotides containing the *sis*-inducible element (SIE) from the c-*fos* gene (m67 mutation) (Figs 3A and S4A)^47^. Notably, both untreated and IL-6-treated nuclear extracts probed with *MMP-1* and *MMP-3* oligonucleotides produced a single band with similar mobility to the SIE-bound STAT-1 homodimer. This *homodimer-like* complex (HDLC) was also found to bind to the novel *MMP-3* SBEs (SBE II/III & SBE IV) (Fig. 3B). The abundance of the HDLC bound to SBE II/III was increased in IL-6-treated cells, and binding to SBE I/AP-1 was enhanced by PMA treatment. Furthermore, binding of the HDLC to the *MMP-3* (SBE I/AP-1) and *MMP-3* (SBE IV) sites became noticeably reduced following IFN-γ treatment, and was blocked entirely by an anti-STAT-1 antibody, but not anti-STAT-3 (Fig. 3C), or anti-STAT-5 antibodies (Supplementary Fig. S4B).

To determine whether AP-1 components were present in the HDLC, extracts were then incubated with either anti-Fos or anti-Jun antibodies. Notably, anti-c-Jun antibody reduced complex binding to the *MMP-1* (SBE/AP-1) probe, and a pan-Fos antibody super-shifted the complex bound to the *MMP-3* (SBE I/AP-1) probe. In addition, binding of the complex to the *MMP-3* (SBE II/III) probe was reduced by anti-c-Jun antibody, suggesting that the HDLC also contained AP-1 (Fos/Jun) components. Notably, in the presence of the non-specific competitor DNA, poly dI:dC (1 μg), binding of the HDLC was only found to occur when the AP-1 binding element was also present (Fig. 3D)^48^. The presence of STAT-1 and AP-1 components binding proximal promoter *MMP* SBEs was confirmed by quantitative chromatin immunoprecipitation (ChIP) analysis (Supplementary Fig. S5).

### STAT-1 not STAT-3 is required for IL-6-mediated induction of *MMP* expression

**Figure 4:**
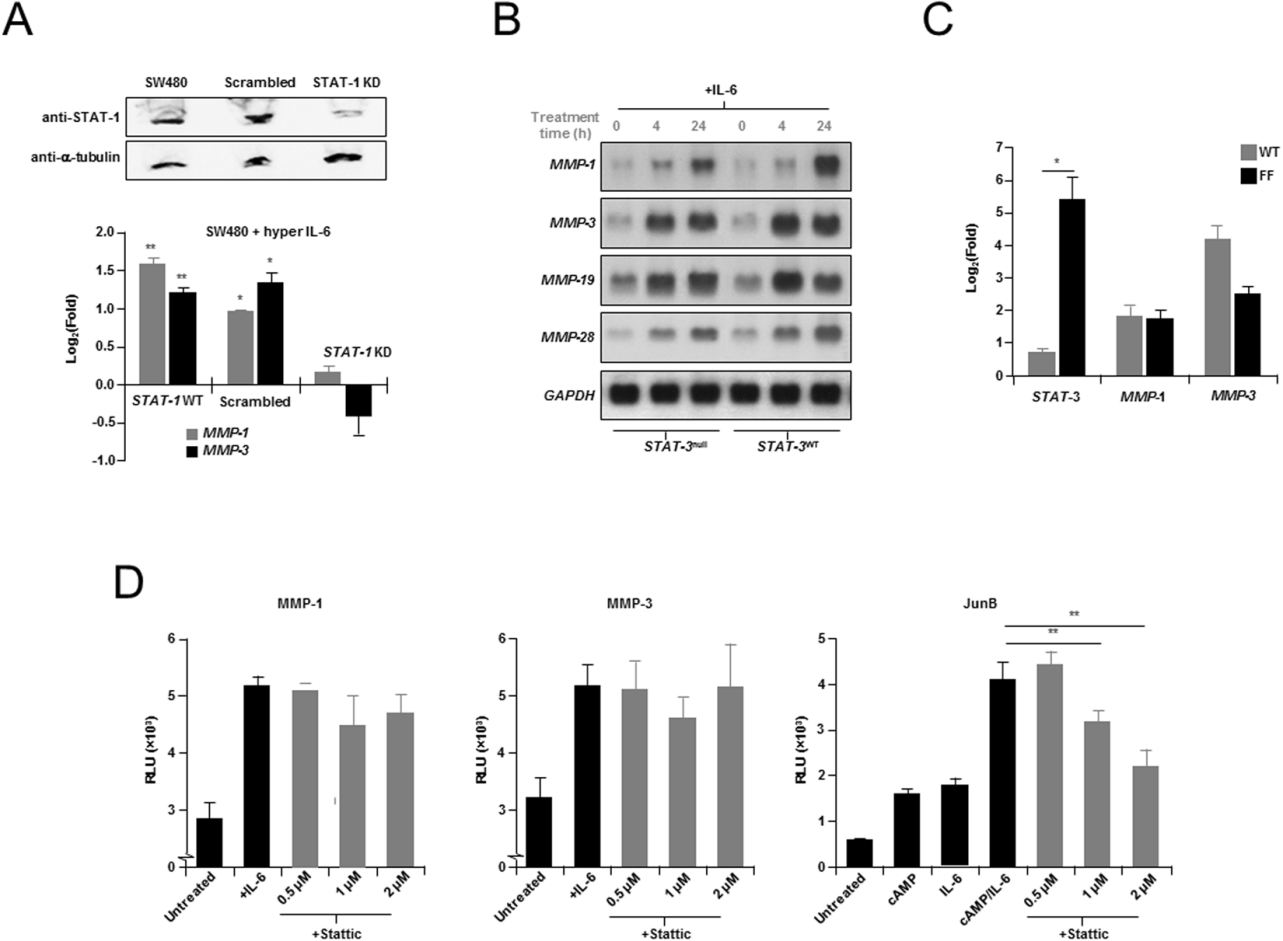
STAT-1 is required for maximal IL-6 induction of *MMPs*. (**A**) Analysis of IL-6-induced *MMP* gene expression in STAT-1 knockdown (KD) human colon cancer cells. *Upper*, Western blot analysis of SW480 STAT-1 KD cells compared with controls, and probed with anti-STAT-1 and anti-α-tubulin antibodies. *Lower*, Wild-type SW480 and SW480 STAT-1 short hairpin shRNA KD cells treated with hyper-IL-6 (20 ng/ml, 18 h) a fusion protein comprising IL-6 and the soluble IL-6 receptor α chain, under conditions of low serum (1.5%). STAT-1 KD has a significantly negative effect on IL-6 induction of MMP-1 and MMP-3. Data is represented as mean Log2(Fold) ± S.E.M., for the difference in mRNA levels between treated and untreated cells (ΔCT_untreated_−ΔCT_treated_), analyzed by independent t test (α = 0.05; **P < 0.01, *P < 0.02).’ (**B**) Northern Blot analyses of human *MMP* gene expression in response to IL-6 in the DLD1 derived *STAT-3* ^null^ A4 colon cancer cell line versus *STAT-3* reconstituted A4 cells. Human colon cancer cells were treated with IL-6 (200 ng/ml), and soluble IL-6R (250 ng/ml) for up to 24 h. Shown, parallel induction of *MMP* mRNA in response to IL-6, in both A4 cells reconstituted with *STAT-3* and *STAT-3* ^null^ A4 cells. (**C**) No enhanced induction of *MMP-1*, or *MMP-3* mRNA levels was observed in STAT hyper-activated gp130Y757F/Y757F (FF) animals following injection of IL-6 (after 90 min), compared with wild-type control animals (n = 5). Also shown, up-regulation of *STAT-3* mRNA, as well as decreased levels of *MMP-3* mRNA following IL-6 treatment in FF animals compared with controls. Data represented as Log_2_(Fold) ± S.E.M. and analyzed by independent *t* test (α = 0.05, *P < 0.05). (**D**) Results of dual-luciferase reporter analysis showing no significant effect on IL-6 (50 ng/ml) and cpt-cAMP (300 μM) induction of *MMP-1* and *MMP-3* full-length promoter constructs pre-treated (1 h) with the STAT-3 inhibitor, Stattic^®^, compared with c-Jun control construct. Constructs were transduced into HepG2 cells and treatment conducted in the presence of 1% serum. Data is represented as mean relative luminescence units (RLU) ± SEM. and analyzed by independent *t* test (α = 0.05, **P < 0.01). For (**A**–**D**), experiments were repeated showing similar results.

Given the positive response of the *MMP* promoter to IL-6 treatment, and the binding of STAT-1 to putative SBEs identified in both the *MMP-1* and *MMP-3* promoters, we next sought to determine whether STAT-1 was required for IL-6 induced *MMP* expression. Firstly, SW480 cells were stably infected with a lentiviral construct containing shRNA targeting STAT-1 (−838)^49, 50^. Control cells were prepared by infecting SW480 cells with a scrambled shRNA construct (Fig. 4A). Notably, STAT-1 knockdown (KD) SW480 cells showed an inability to induce *MMP-1* or *MMP-3* mRNA following treatment with hyper-IL-6^51^; the recombinant form of IL-6, which contains both IL-6 and soluble IL-6 receptor, to ensure maximum activation (Fig. 4A). This contrasted with parental SW480 cells which showed a 3-fold (P = 0.0002) and 2.3-fold (P = 0.008) increase in *MMP-1* and *MMP-3* mRNA levels, respectively. Scrambled controls showed similar levels of hyper-IL-6-mediated *MMP-1* and *MMP-3* induction to that observed in parental controls (P = 0.032 and P = 0.01, respectively). This result is consistent with a requirement for STAT-1 in the IL-6-mediated activation of *MMP-1* and *MMP-3* genes in colon cancer.

Although direct binding studies did not suggest a role for STAT-3 in *MMP* promoter activation we next examined the requirement, if any, for STAT-3 in IL-6 activation of *MMP-1* and *MMP-3* expression. To do this we first compared IL-6 treated DLD1 derived *STAT-3* ^null^ A4 colon cancer cells versus *STAT-3* reconstituted A4 cells^52^. In this case, the absence of STAT-3 had no obvious effect on IL-6 induction of *MMP-1*, *MMP-3*, *MMP-19* or *MMP-28* over a 24 hour time-course (Fig. 4B). We next examined *MMP* expression in the gp130 mouse model, which shows hyper-activation of both STAT-1 and STAT-3 in response to IL-6^53^. Notably, following direct injection of IL-6 into the livers of these animals no significantly enhanced expression was identified in MMP expression in the tissues of STAT-3 hyper-activated homozygous (FF) animals when compared with wild-type controls (Fig. 4C).

The lack of dependence on STAT-3 for IL-6-induced *MMP* gene activation was further supported by experiments using the small molecule inhibitor of STAT-3 (Stattic^®^)^54^. Administration of Stattic^®^ showed no significant effect on IL-6-induction of *MMP-1* and *MMP-3* proximal promoter constructs, compared with the STAT-3 inducible c-Jun control (Figs 4D and S6).

## Discussion

In this study we have used comparative gene expression analysis and promoter mapping to demonstrate a direct link between *IL-6* and *MMP* expression in colon cancer, via STAT-1 and novel non-canonical SBEs identified in the *MMP-1* and *MMP-3* proximal promoters. Firstly, in a patient matched study comparing tumor tissue with mucosa we determined that: (i) expression of cytokines (*IL-6* and *LIF*) clustered with proteases, including *MMP-1*, *MMP-3* and *MMP-7*; (ii) correlation was generally linked to pathophysiology of the tumor with higher levels in more restricted Duke’s B tumors compared with invasive Duke’s C tumor; and (iii) *MMP-1* and *MMP-3* were co-regulated with previously identified downstream targets of IL-6 in colon cancer cell lines (Fig. 1). While the reason for decreased expression in Duke’s C tumors is unclear the close correlation between IL-6 and *MMP* levels *in vivo* and *in vitro*, as well as the presence of potentially IL-6 responsive SBEs in the *MMP* promoters, did suggest a high degree of co-regulation between IL-6 and *MMPs*, which we examined further.

Notably, while known ISGs were positively regulated by IFN-γ in colon cancer cell lines, MMP-1, which also contains a GAS-like SBE in its proximal promoter, was down-regulated by IFN-γ. At least one previous report has shown that this element is responsive to IL-6/OSM/LIF^25^. Through reporter analysis we confirmed that this proximal SBE/AP-1 element was responsive to IL-6. Interestingly, *MMP-3*, which is also responsive to IL-6 and co-regulated with IL-6 in our study, has previously been shown to be regulated via STAT-3 through distal promoter elements^26^. After analysis of the proximal promoter of *MMP-3*, we identified five new non-canonical SBEs in the *MMP-3* promoter, including a tandem SBE/AP-1 element, which were also responsive to IL-6 (Fig. 2). When direct binding studies were conducted they provided further evidence for the constitutive and enhanced binding of a protein complex (HDLC) to these SBEs, which shifted to the same size as the classical IFN-γ activated STAT-1 homodimer^14, 15^. HDLCs bound to these elements were found to contain STAT-1 protein, and either Fos (*MMP-3)* or c-Jun (*MMP-1*), and were negatively regulated by IFN-γ (Fig. 3). Notably, sIL-6R, or the hyper-IL-6 protein did not significantly alter IL-6 induction of *MMP* promoter elements (Figs 2C and S3B), or HDLC binding (Supplementary Fig. S4A), suggesting that endogenous sIL-6R was sufficient to ensure maximum promoter activation in response to IL-6.

Through the use of shRNAi knockdown we have also showed that STAT-1 is required for maximal activation of *MMP-1* and *MMP-3* in response to IL-6, while knockout of STAT-3 in a colon cancer cell line had little or no effect on IL-6 induction of *MMP-1* and *MMP-3*. We also demonstrated that neither hyperactivation of STAT-3 in the gp130 mice, or inhibition of STAT-3 protein using the small molecule inhibitor Stattic^®^, did not significantly affect IL-6 activation of *MMP-1* and *MMP-3*. These results collectively suggest that STAT-1, and not STAT-3, is required for IL-6 activation of *MMP* gene expression via proximal promoter SBEs (Fig. 4).

The discovery of a STAT-1 containing complex that cooperates with AP-1 to regulate MMPs through GAS-like SBEs in the *MMP-1* and *MMP-3* proximal promoters is novel. However, similar promoter regulatory complexes have previously been described including: (i) an IFN-γ-inducible complex comprising STAT-1 and c-Fos that binds to adjacent GAS and AP-1 binding elements in the human *Nitric Oxide Synthase (NOS) 2* promoter^55^, and (ii) an IL-12 and IL-18 responsive c-Jun-STAT-4 complex that binds to an AP-1 binding element in the IFN-γ gene (*IFNG*) promoter^56^. Interestingly, in this last case only c-Jun appears to contact DNA directly, and promoter binding is dependent on the presence of STAT-4. In our study, while c-Fos was constitutively bound to the *MMP-3* promoter prior to the addition of IL-6/PMA, it may be that PMA treatment also induced more STAT-1 to bind, given that PMA has previously been shown to activate JAK/STAT signaling^57^. We also found that STAT-1 binding to the *MMP-3* proximal promoter was reduced by treatment of cells with IFN-γ.

**Figure 5:**
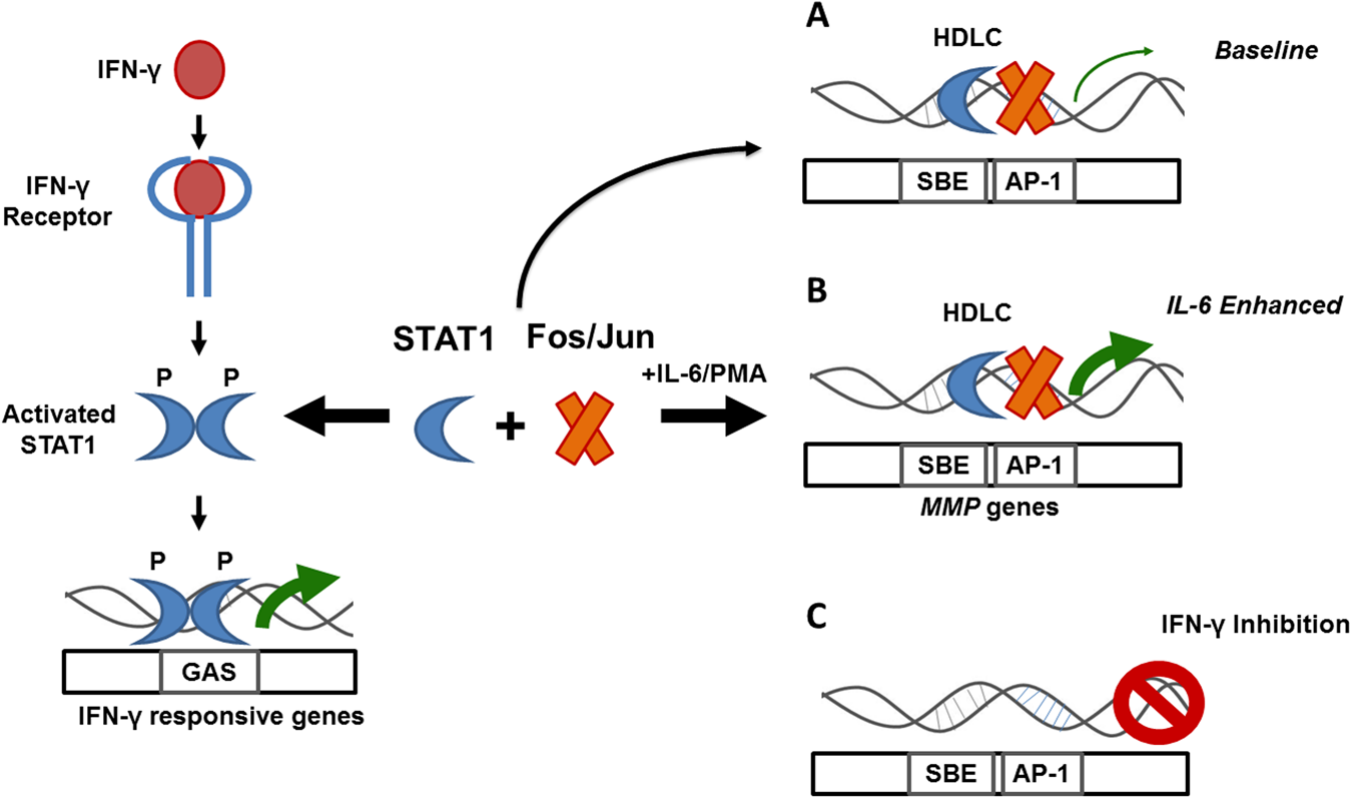
Proposed model for the regulation of *MMPs* by IL-6/PMA and IFN-γ. Fos/Jun and non-phosphorylated STAT-1 bind constitutively to AP-1 and SBEs elements in the *MMP-1* and *MMP-3* proximal promoters, maintaining a basal level of gene expression (**A**). In the presence of IL-6 or PMA, MMP expression is enhanced (green arrow) (**B**). In response to IFN-γ, STAT-1 is phosphorylated, increasing its affinity for binding to GAS elements in the promoters of IFN-γ responsive genes, displacing STAT-1 from the HDLCs in the *MMP* gene promoters, resulting in a reduction in expression of these genes (**C**).

Previous studies have also shown suppression of *MMPs* in response to IFN-γ, such as *MMP-9*, which like *MMP-1* and *MMP-3*, also contains AP-1 sites (two) and putative SBEs^58–60^, and has been shown to be repressed following IFN-γ treatment in astrocytes, fibrosarcomas, and monocytes. In all of these cases, loss of *MMP* gene activity following IFN-γ treatment is likely to relate to changes in the phosphorylation status of STAT-1 post-activation, and suggests a possible novel mechanism of cytokine regulation of gene expression. In contrast to IFN-γ responsive genes such as *IRF-1* ^15^, which contain canonical GAS elements activated by P-STAT-1, STAT-1 regulation of *MMP* SBEs does not appear to be dependent on IFN-γ-mediated tyrosine phosphorylation. Instead, activation of STAT-1 by IFN-γ may lead to dimerization of STAT-1, a breakup of the U-STAT-1/AP-1 complex bound to the GAS-like SBEs present in the *MMP-1* and *MMP-3* promoters, as well as a reduction in gene promoter activity (Fig. 5). In this way, STAT-1 hyperactivation in the gp130 (FF) animals, which leads to a decrease in *MMP-3* levels in response to IL-6 (Fig. 4C), may also be explained by reduced availability of U-STAT-1 for binding to the HDLC.

Such a mechanism for explaining promoter regulation by STATs is supported by previously published data. Indeed it has been shown that STAT-1 exists in different homodimeric confirmations depending on whether, or not﻿, it is tyrosine phosphorylated^61^, and unphosphorylated homodimer (U-STAT-1) differs in binding specificity (and genes induced) to those of the tyrosine phosphorylated P-STAT-1 homodimeric form activated by IFNs^62^. The fact that IFN treatment leads to a reduction in MMP expression and promoter activity, and that P-STAT-1 is not a significant contributor to IL-6 induced MMP expression in the hyperactivated model (FF) of STAT signalling (Fig. 4C), strongly suggests that U-STAT-1 plays an important role in contributing to the formation of the HDLC and regulation of noncanonical SBEs present in the proximal *MMP* promoters.

While the involvement of U-STAT-1 in regulating gene expression has been shown by others^16, 62, 63^, to the best of our knowledge this is the first report elucidating a novel STAT-1 binding complex to noncanonical SBEs and whose binding is constitutive, enhanced by IL-6, and inhibited by IFN-γ treatment. Furthermore, sensitivity to the presence of poly-dI:dC displayed by STAT-1 HDLC binding to the SBEs without the AP-1 motif suggests that formation of this complex is not robust and requires AP-1 components to facilitate STAT-1 binding. Intriguingly, despite previous reports showing primarily STAT-1, but also STAT-3 binding to the AP-1 proximal SBE element in the *MMP-1* promoter^25^, as well as STAT-3 regulating *MMP-3* via distal promoter elements^26^, we did not observe any STAT-3 dimer bands binding the *MMP-3* SBE/AP-1 motif, and *MMP* induction by IL-6 was not significantly affected in Stat-3^*null*^ cells. All of this evidence is consistent, and indicates that STAT-3 activation is not a precondition for IL-6 induction of either *MMP-1* and/or *MMP-3*.

## Conclusion

In this study, we present data from comparative biopsy analysis, confirming co-regulation of MMPs with inflammatory cytokines, such as IL-6, in colon cancer. We also show the results of the analysis of novel GAS-like SBEs in the proximal promoter of the *MMP-3* gene, which bear sequence similarity to an element previously identified within the *MMP-1* promoter^25^. Similar to the *MMP-1* SBE, the proximal *MMP-3* GAS-like SBEs bound a complex (HDLC) that contained both STAT-1, and components of AP-1 (c-Fos/Jun), but not STAT-3. Furthermore, while STAT-3 may play a role in maximal induction of *MMP-3*, we show that ablation or inhibition of STAT-3 does not substantially affect the IL-6 induction of *MMPs*. Instead, IL-6-mediated induction of *MMPs* was substantially affected by knockdown of STAT-1. Uniquely, for GAS-like SBEs, we also provide evidence for negative regulation of *MMPs* by IFN-γ. Collectively, the results suggest a mechanism of regulation of MMPs by IL-6 that relies on enhancing STAT-1 binding in a HDLC with AP-1 components, and which is negatively regulated following activation/phosphorylation of STAT-1 by IFN-γ. This does not contradict previous work^26, 29^, which has shown a requirement for STAT-3 activation of distal SBEs in maximal induction of *MMPs* by IL-6. Instead, functional STAT-3 may not be a precondition for IL-6-mediated *MMP* induction. This has important clinical implications, especially for the development of drugs (eg. Stattic^®^) that target STAT-3 activation and the expression of STAT-3 target genes^31^. Our findings also provide evidence to support future studies designed to more fully define the role of U-STAT-1 in inflammatory cytokine-mediated gene regulation and cancer malignancy.

## Methods

### Cell lines and Treatment

The human colorectal carcinoma cell lines HT29, SW480, LISP-1, LIM1215, HCT116, and LS174T, as well as the hepatocyte-derived HepG2 cell line, were obtained from the American Type Culture Collection (ATCC) (Manassas, VA, USA). Cells were grown in either RPMI 1640 (Sigma-Aldrich, St. Louis, MO) or DMEM (Sigma-Aldrich) under either standard serum [10% fetal calf serum (FCS), 5% CO_2_] or low serum (<1% FCS) growth conditions. Human DLD1 colon carcinoma cells used to derive a Stat-3^*null*^ sub-line were also obtained from the ATCC and were grown in McCoy’s 5A medium with L-glutamine (Hyclone, Logan, UT). Unless otherwise indicated, media were supplemented with 10% FCS, penicillin (100 U/ml), and streptomycin (100 μg/ml) (Gibco-BRL, Carlsbad, CA). Cytokines used in this study include human type-I IFN (-β) (Biogen, Cambridge, MA), and type-II (-γ) (Hoffman-La Roche, Basel, Switzerland), as well as IL-6 (Peprotech, Rocky Hill, NJ), hyper-IL-6 (kind gift from Prof. Stefan Rose-John) and Leukaemia Inhibitory Factor (LIF) (Peprotech). Notably, while all cell lines were found to express gp-130 and IL-6 receptor, both of which are required for maximal induction of IL-6 target genes, addition of sIL-6R, and or the recombinant hyper-IL6 (a fusion of IL-6 & IL-6R) was used to ensure maximal IL-6/STAT activation^51^, and was used as indicated. Cells were also treated with the activator of AP-1, phorbol 12-myristate 13-acetate (PMA) (Sigma-Aldrich), at indicated concentrations.

### Patient samples

Tumor tissue and distal normal colonic mucosa from the primary surgical margins were obtained from patients who had undergone corrective surgery. The mean patient age was 74. All procedures were conducted under the guidelines outlined by the National Health & Medical Research Council, Australia, and approved by Linkoping University Hospital (Sweden), University of NSW (Australia) and Griffith University (Australia), Human Research Ethics Committees. Written informed consent was obtained from all patients and the healthy volunteer. Patients were chosen based on their clinical diagnosis.

Upon collection each sample was snap frozen and a representative region embedded in paraffin for histopathological examination. Samples were first assessed by PCR-single stranded conformational analysis (SSCA) and direct sequencing for point mutation analysis^64^. Total RNA was isolated (RNeasy^®^ Mini Kit, Qiagen, CA) and first strand synthesis carried out using 1–5 μg of total RNA, Superscript^TM^ (Invitrogen Corp., San Diego, CA). All reactions contained 1 × SYBR Green I mix (ABI) and custom primers (Supplementary Table 1). 18SrRNA was used as a normalization reference and data analyzed, as previously described^65^.

### Dual-reporter assays

Transfections were performed using previously published methods^44^. Cultures were grown to subconfluence in antibiotic-free media (10% FCS). Each transfection contained a pGL3-MMP promoter construct (1 μg), 1.5 μl jetPEI^TM^ reagent (Polyplus Transfection, Illkirch, France), and 10 ng phRL-SV40, encoding the internal standard *Renilla* luciferase. Following transfection, cells were treated with: IL-6, soluble IL-6Rα (Peprotech), PMA and/or human IFN-γ. Luciferase activity was recorded using the BMG FLUOstarOPTIMA microplate reader (BMG Labtech, Melbourne, Australia) using *Renilla* luciferase as a normalization standard.

### Direct DNA binding (EMSA) studies

Nuclear extracts were prepared using the detergent-free procedure described previously^66^. Oligonucleotide (Supplementary Table 3) were end labeled using a dNTP mix containing [α-^32^P] dCTP (Amersham, Piscataway, NJ). Nuclear extracts (5 μg) were incubated (20 min, RT) with probes in binding buffer (20 mM HEPES pH 7.8, 1 mM MgCl_2_, 0.5 mM DTT, KCl [40–80 mM] & 5% glycerol), and protein/DNA complexes resolved by PAGE (5% glycerol). Antibodies used for supershifting included anti-STAT-1 (polyclonal; #9172; Cell Signaling Technology Inc, Beverly, MA), anti-STAT-3 (polyclonal; #9132; Cell Signaling Technology Inc), anti-STAT-5 (polyclonal; #9310; Cell Signaling Technology Inc), anti-c-Jun (polyclonal; #9162; Cell Signaling Technology Inc) and anti-pan-Fos (K-25; #sc-253X; Santa Cruz Biotech Inc, Santa Cruz, CA).

### Production of STAT-1 knockdown SW480 colon cancer cell line

293T cells were transfected with 15 μg pCMV-dR8.74 (gag-pol), 6 μg pMD-2G (VSV-G env) and 20 μg pLKO.1-puro containing STAT-1 (-838) or scrambled short hairpin RNA (shRNA). Viral supernatants were harvested after 48 h and concentrated by centrifugation. SW480 cells were infected with lentivirus particles in the presence of 8 μg/ml polybrene and selected with 2 μg/ml puromycin. Scrambled knockdown cells were pooled while STAT-1 knockdown cells were clonally selected. Antibodies used for western blotting included anti-STAT-1 (monoclonal, 9H2; #9176; Cell Signaling Technology Inc) and anti-α-tubulin (monoclonal, DM1A; #T9026; Sigma-Aldrich).

### Data analysis

All experiments were repeated at least once, and included at least three to five experimental replicates for purposes of statistical analysis. Unless otherwise stated data was analyzed using GraphPad Prism™. The differences between control and test data was determined by an independent samples *t* test (α = 0.05). Hierarchical clustering was carried out using the nearest-neighbour method. Either mean fold or median (box plots) have been used to represent relative difference in gene expression between tumor and normal mucosa 2^(ΔCTcontrol−ΔCTtest)^, and for promoter response to cytokine treatment (RLU_treated_/RLU_untreated_).

### Data Availability

All data generated or analysed during this study are included in this published article (and its Supplementary Information files), or available from the corresponding author on reasonable request.

## Electronic supplementary material

Supplementary Information
