# Supplementary material for: Novel STAT binding elements mediate IL-6 regulation of MMP-1 and MMP-3
Source: Sci Rep. 2017 Aug 17;7:8526. doi: 10.1038/s41598-017-08581-y (PMC5561029; doi:10.1038/s41598-017-08581-y)
Supplement: Supplementary file 1 — Supplementary Information [file 41598_2017_8581_MOESM1_ESM.pdf]

Novel STAT binding elements mediate IL-6 regulation of MMP-1 and MMP-3.

Samuel J. Cutler[1], James D. Doecke[1], Ibtisam Ghazawi[1], Jinbo Yang[2], Lyn R. Griffiths[3], Kevin J. Spring[4,5], Stephen J. Ralph[1*#] , Albert S. Mellick[1,4,5*#]

[1]School of Medical Science, Griffith Institute of Health & Medical Research, Griffith University, Gold Coast Campus, QLD, Australia.

[2]Department of Molecular Genetics, Lerner Research Institute, 9500 Euclid Avenue, Cleveland, Ohio 44195, USA.

[3]Institute for Health & Biomedical Innovation, Queensland University of Technology, 60 Musk Avenue, Kelvin Grove, QLD 4059, Australia.

[4]School of Medicine, Western Sydney University, Locked Bag 1797, Penrith NSW 2751, Australia.

[5]Ingham Institute for Applied Medical Research, South Western Sydney Clinical School UNSW & CONCERT Translational Cancer Research Centre, 1 Campbell Street, Liverpool, NSW 2170, Australia.

*Correspondence and requests for materials should be addressed to either: s.ralph@griffith.edu.au (S.J.R.) or a.mellick@unsw.edu.au (A.S.M.).

[#]These authors contributed equally to this work.

Funding: This research was supported by grants held by A.S.M and S.J.R: Australian Research Council (Grant No 0988602), Queensland Cancer Fund (Grant No 277014) and National Health & Medical Research Council (Grant No 1011114). S.J.C. and I.G. were supported by Australian Postgraduate Research Awards.

Competing interest: The authors declared that no competing interests exist.

Supplementary Methods

Construction of reporter vectors. Primers for amplification of the 548bp fragment from the *MMP-1* promoter (forward: 5'-CAATAGGGTACCAGGCAGCTTA-3' & reverse: 5'-AGTGA**AA**GCTTCCCAGCCTCTT-3'), *MMP-3* promoter 607 bp (forward: 5'-CCCATGAG**C**T**C**TTCCTCCTCAA-3' & reverse: 5'-GCTC**A**A**GC**TTGTCTCTATGCCTT-3'), were designed with specific alterations that resulted in the generation of *Sac*I and *Hin*dIII sites (underlined), used for cloning into pGL3-basic (Promega, Madison, WI). For specific analysis of MMP-3 SBEs, progressively shorter regions were also cloned, using the same reverse primer (5'-GAGCG**A**AGCTTTTAAAGAGTGAC-3'), but different forward primers: SBE I/II/AP-1: 5'-CTTTGA**GC**T**C**GGAATGTTTGGAA-3'; SBE I/II/III/AP-1 5'-AAAGGAGCTCGTATCATCCTACT-3'; SBE I/II/III/IV/AP-1 5'-TTTTGAGCTCTACCAAGACAGCA-3'; and SBE I/II/III/IV/V/AP-1 5'-CATAAGAGCTCTAGTGAATTCCAG-3'.

Chromatin Immunoprecipitation (ChIP) Assays. Where indicated before fixation (1% formaldehyde; 15 min) cells were treated with PMA (100 nM, 90 min) and/or IL-6 (50 ng/ml, 20 min). For ChIP, cells were 125 mM glycine, washed (PBS) and detached in the presence of protease inhibitors: aprotinin (15K U/ml), leupeptin (3 μg/ml), PMSF (300 μM) and pepstatin (1 μg/ml). Cell pellets were then resuspended in sonication buffer (1 % Triton X-100, 0.1 % sodium deoxycholate, 50 mM Tris-HCl pH 8.1, 5 mM EDTA, 150 mM NaCl, protease inhibitors as above) and sonicated on ice for 8 bursts of 10 sec. Cell debris was removed by centrifugation (15000 g, 15 min, 4˚C). Supernatants were pre-cleared with 40 μl protein G sepharose (50 % slurry). Sheared herring sperm DNA (2 μg) was then added for a further 2 h. Precipitates were washed sequentially for 10 min with sonication buffer, twice with buffer II (1 % Triton X-100, 0.1 % sodium deoxycholate, 50 mM Tris-HCl pH 8.1, 5 mM EDTA, 500 mM NaCl, protease inhibitors), once with buffer III (250 mM LiCl, 0.5 % Nonidet P-40, 0.5 % sodium deoxycholate, 10 mM Tris-HCl pH 8.1, 1 mM EDTA, protease

inhibitors), and three times with TE buffer containing protease inhibitors. Bead precipitates were eluted twice with 150 µl elution buffer (1 % SDS, 100 mM NaHCO3) and samples incubated (65˚C, 4 h) with 0.3 M NaCl and 3 ng RNase A, following which the samples were left overnight at 45˚C with 75 µg proteinase K. DNA was then purified and qPCR conducted, following methods described. Data is presented as percentage (%) of total input DNA bound.

Antibodies used for ChIP included anti-STAT-1α (C-24; #sc-345X; Santa Cruz Biotech Inc) and anti-pan-Fos, as above. ChIP assays were conducted using primers to amplify a 188 bp sequence in the *MMP-3* promoter as follows: *forward*; 5'-TCCTCTACCAAGACAGGAAGC-3' and *reverse*; 5'-TTTGTTTGGATCACCCGCAGC-3'. To obtain extracts, cells were grown to 60 % confluence and cultured for a further 24 h in reduced serum (1 %), prior to cytokine treatment. Following fixation and binding, DNA was purified and Q-PCR analysis conducted using SYBR green I following methods described above. The amount of precipitated DNA was calculated as a percentage of input DNA.

Production of the A4 STAT-3 null colon cancer cell line and northern blotting analysis. The *STAT-3* gene was knocked out in DLD1 cells by homologous recombination to make the STAT-3-null A4 cell line using pAAV-Neo-loxP similar to the methods described previously[52]. A4 cells were then reconstituted to re-express wild-type STAT3 by stable transfection of a retroviral expression vector, which was generated by inserting the human STAT-3 cDNA into the *Hin*dIII site of pLEGFP-N1 (Clontech, Palo Alto, CA). Stably transduced A4 cell pools were selected following treatment with G418 (500 µg/ml). Cells were either treated with IL-6 (200 ng/ml) together with IL-6 soluble receptor (250 ng/ml) or left untreated. Probes used for analysis of human *MMP* gene expression were generated by RT-PCR by using gene specific primer sets. Primer sequences were as follows: 5'-ATGGAGGCGCTAATGGCCCGGGGCGCGC-3' (forward) and 5'-GGGTTGCCGCAGCGTGGCTTCCGCA-3' (reverse) for the *MMP-1* gene corresponding to 1-615 bp of its coding domains; 5'-ATGAAGAGTCTTCCAATCCTAC-3' (forward) and 5'-CATAGGCATGGGCCAAAACAT-3' (reverse) for the *MMP-3* gene corresponding to 1-616 bp of its coding domains; 5'-ATGAACTGCCAGCAGCTGTGGC-3' (forward) and 5'-GAACAGTACGAGCTTTGGCGG-3' (reverse) for the *MMP-19* gene corresponding to 1-620bp of its

coding domains; 5’-ATGGTCGCGCGCGTCGGCCTC-3’ (forward) and 5’-CTTACGCCTCATTTTGGTCCGGTG-3’ (reverse) for the *MMP-28* gene corresponding to 1-600 bp of its coding domains. PCR products were purified by using MinElute PCR Purification Kits (Qiagen, Valencia, CA) and monitored by DNA sequencing for fidelity and integrity. 100 ng of each DNA fragment was used to synthesise ^{32}P-labeled probes with the megaprimer labelling kit (Amersham Biosciences, Piscataway, NJ). 20 μg of total RNAs were used for northern blotting. Templates for human GAPDH cDNA as control were also prepared by RT-PCR as well. Signals were normalized for loading by comparing the intensities of *GAPDH* mRNA by re-probing the membranes analyzed for *MMP* mRNA induction.

Supplementary Figure Legends

Supplementary Figure S1. (**A**) Correlation between levels of *Interleukin (IL) 6* and the family member *Leukaemia Inhibitory Factor* (*LIF)*, with *MMP-1, MMP-3*, and *MMP-7* gene activity in colorectal cancer tissue. Data was analyzed by Spearman’s ρ correlation (α = 0.05). The P-value for each association is indicated numerically and the correlation coefficient (ρ value) is indicated by color. The power for the study was calculated to be 99.89 % (n = 52; 0.25 < ρ < 0.75 for the Null Hypothesis; 95 % Confidence). (**B**) Relative levels of mRNA for *IL-6* and *LIF*, as well as their receptor subunits: *gp130, LIF receptor (LIFR) & IL-6 receptor (IL-6R*), in HT29, SW480 and LS174T colon cancer cell lines. Relative expression is shown as mean (ΔCT) ± SEM. (**C**) Expression changes of IL-6 regulated genes (*STAT-1, IRF-1, STAT-3* & *BCLXL*), compared with *MMP-1* in SW480, HCT116, LISP-1 and LIM1215 colon cancer cell lines, following treatment with Interferon (IFN)-γ (500 U/ml, 18 h). Change in expression is presented as mean Log_2(Fold) ± SEM. Notably, IFN-β treatment showed no consistent trend across cell lines in *MMP-1* activation (*not shown*). For (**B**) & (**C**) analysis was conducted by Independent *t* test (*P < 0.05), and experiments repeated showing similar outcomes.

Supplementary Figure S2. Shown, alignment of the *MMP-1* (**A**) and *MMP-3* (**B**) proximal promoters from mouse, rat, canine, chimp and human, revealed highly conserved STAT binding

elements (SBEs) (Shading) and AP-1 recognition sequences (Boxed), proximal to the TATA start site of transcription (Boxed).

Supplementary Figure S3. (A) Shown, comparison of the constitutive and IL-6-induced levels of luciferase expression produced from the *MMP-1* prom (549 bp) construct under low (1%) serum concentrations transfected into SW480 colon cancer cell line. Following IL-6 induction (10 ng/ml, 18 h), dual-luciferase assays were performed. **(B)** Shown, comparison of IL-6-induced levels of luciferase expression produced from the *MMP-3* prom (607 bp) construct, with and without sIL-6R, in SW480 colon cancer cells. Following addition of either IL-6 (10 ng/ml, 18 h) of sIL-6R (50 ng/mL), dual-luciferase assays were performed. For (A) & (B), data is represented as mean relative light units (RLU) ± SEM. and was analyzed by Independent *t* test ($\alpha = 0.05$, $*P < 0.05$). Experiments were repeated showing similar outcomes.

Supplementary Figure S4. (A) Extracts from HepG2 cells grown in low serum conditions (1 %) and treated with: (**A**) IFN-γ (1000 U/ml, 1 h), IL-6 (50 ng/ml, 15 min), sIL-6R (50 ng/ml, 15 min) or PMA (10 nM, 15 min) and incubated with SIE probe, and/or *MMP-1* SBE/AP-1 probe; and (**B**) IFN-γ (1000 U/ml, 1 h) or IL-6 (50 ng/ml, 5 min) incubated with SIE probe, and/or *MMP-3* SBE I proximal promoter probe. Shown, STAT homer dimer-like complexes (HDLC) binding to *MMP-1* (SBE/AP-1) and MMP-3 (SBEI no AP-1) proximal promoter elements. For (A) & (B), antibodies were added to the reaction either: (α) after adding the probe (post-incubation) or (β) prior to adding the probe (pre-incubation). Each experiment was repeated showing similar outcomes.

Supplementary Figure S5. (**A**) Shown, chromatin extracts from SW480 cells transfected with *MMP*-3 STAT-binding region, treated with IL-6 (50 ng/ml, 20 min) and PMA (100 nM, 90 min) were amplified by PCR, compared with untreated and IgG controls. *Shown*, specific Chromatin Immunoprecipitation (ChIP) reaction products obtained from the SBE containing region of the

MMP-3 promoter with anti-STAT-1 and anti-pan-Fos sera, but not from the control region that did not contain the SBEs. (**B**) Results of quantitative analysis of ChIP reaction products showing increased binding of STAT-1 following PMA/IL-6 treatment. Data represented as mean % of input DNA obtained with anti-STAT-1 sera after treating SW480 cells with IL-6/PMA ± SEM, and was analyzed by Independent *t* test ($\alpha = 0.05$, *$P < 0.01$). For (A &B), the experiment was repeated showing similar outcomes.

Supplementary Figure S6. Shown, time course of induction by IL-6 from luciferase reporter vectors containing (**A**) *JunB* SBE and (**B**) MMP-3 prom 607 bp. HepG2 cells cultured in the presence of 1 % foetal calf serum (FCS) were transfected with 0.4 μg reporter vector DNA using FuGENE® HD transfection reagent. Cells were treated with IL-6 (50 ng/ml) for the time periods shown. For optimal induction of the *JunB* construct, cells were concurrently treated with IL-6 and cpt-cAMP (300 μM). Following IL-6 treatment, dual-luciferase assays were performed. Luciferase activity is shown as fold ± SEM, relative to the non-IL-6-induced (control).

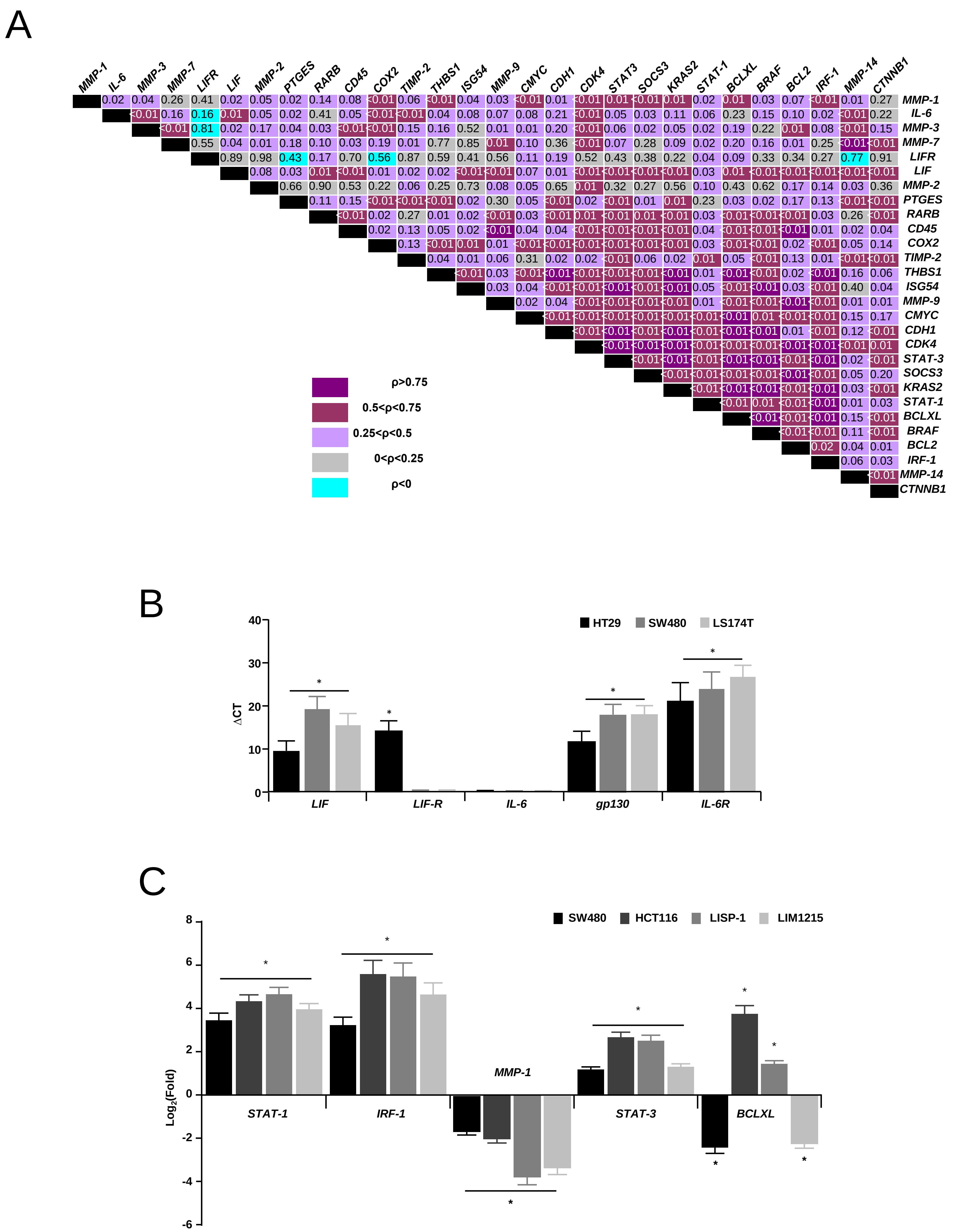
A
MMP-1 IL-6 MMP-3 MMP-7 LIFR LIF MMP-2 PTGES RARB CD45 COX2 TIMP-2 THBS1 ISG54 MMP-9 CMYC CDH1 CDK4 STAT3 SOCS3 KRAS2 STAT-1 BCLXL BRAF BCL2 IRF-1 MMP-14 CTNNB1
MMP-1 IL-6 MMP-3 MMP-7 LIFR LIF MMP-2 PTGES RARB CD45 COX2 TIMP-2 THBS1 ISG54 MMP-9 CMYC CDH1 CDK4 STAT-3 SOCS3 KRAS2 STAT-1 BCLXL BRAF BCL2 IRF-1 MMP-14 CTNNB1
ρ>0.75
0.5<ρ<0.75
0.25<ρ<0.5
0<ρ<0.25
ρ<0
B
HT29 SW480 LS174T
ΔCT
40
30
20
10
0
LIF LIF-R IL-6 gp130 IL-6R
C
SW480 HCT116 LISP-1 LIM1215
Log2(Fold)
8
6
4
2
0
-2
-4
-6
STAT-1 IRF-1 MMP-1 STAT-3 BCLXL

A

			AP-1			SBE			TATA	
Human	AGGATGTT	ATAAAGCA	**TGAGTCA**	GACAG	CCTCTGGC	**TTTCTGGAA**	GGG	CAAGGACTCTA	TATATACA	G
Chimp	AGGATGTT	ATAAAACA	**TGAGTCA**	GACAG	CCTCTGGC	**TTTCTAGAA**	GGG	CAAGGACTCTA	TATATACA	G
Mouse	AGGATGTT	GTCAACCA	**TGAGTCA**	CATAG	CCTCTGGC	**TTTCTAGAA**	GTA	CAAGAAGTCTC	TATATAAA	G
Rat	AGGATGTT	GTCAACCA	**TGAGTCA**	CGTAG	CCTCTGGT	**TTTGTAGAA**	GTA	---GAAATCTC	TATATAAA	G
Canine	AGGATGTT	ATGAAACG	**TGAGTCA**	GCCAG	CCTCCGGC	**TTTCTGGAA**	GTG	TGGGT---CTG	TATATAAA	A

B

		SBE V			SBE IV	
Human	CAA---TT	**TTTCCAGAA**	--GAAAA…	…AGCAC	**TTCCTGGAG**	ATTAATC…
Chimp	CAA---TT	**TTTCCAGAA**	--GAAAA…	…AGCAC	**TTCCTGGAG**	ATTAATC…
Mouse	TAA---AT	**TTTCCAAAG**	TGGAAAA…	…AGCAT	**TTCCTGGAG**	ATTAATC…
Rat	TAC---AT	**TTTCCAAAG**	TAGAAAA…	…AGCAT	**TTCCTGGAG**	ATTAATC…
Canine	CACTTTTT	**TTTCCATAA**	--TAAAA…	…AGCAT	**TTCCTGGAG**	CTTAATC…

		SBE III		SBE II	
Human	…ATCC	**TACTTTGAA**	TTTGGAA	**TGTTTGGAA**	ATGGTCCTGCTGCCAT
Chimp	…ATCC	**TACTTTGAA**	TTTGGAA	**TGTTTGGAA**	ATGGTCCTGCTGCCAT
Mouse	…ACCC	**TACTCTGA-**	TTTTTAA	**TTTTTGGAA**	GTGGTCC------CAT
Rat	…ACTC	**T--TCTGA-**	TTTTTAA	**TTTTTGGAA**	ATGGTCC------CAT
Canine	…ATCC	**TACTTTGGA**	TTTGGAA	**T-TTTGGAC**	ATGGTCCTGCTGCCAC

	SBE I		AP-1			TATA
Human	**TTGGATGAA**	AGCAAGGA	**TGAGTCA**	AGCTGC…	…CACTC	TTTAAA
Chimp	**TTGGATGAA**	AGCAAGGA	**TGAGTCA**	AGCTGC…	…CACTC	TTTAAA
Mouse	**TTGGATGGA**	AGCAATTA	**TGAGTCA**	GTTTTC…	…CACTC	TATAAA
Rat	**TTGGATGGA**	AGCAATTA	**TGAGTCA**	GTTTGC…	…CACTC	TATAAA
Canine	**TCGGATGAA**	AGCAATCA	**TGAGTCA**	AGCTGC…	…CGCCC	TATAAA

Cutler *et al*. FigS3

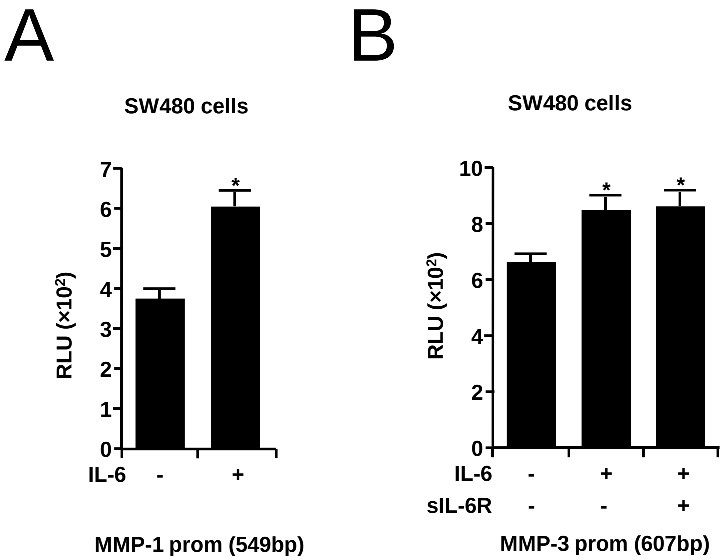

A

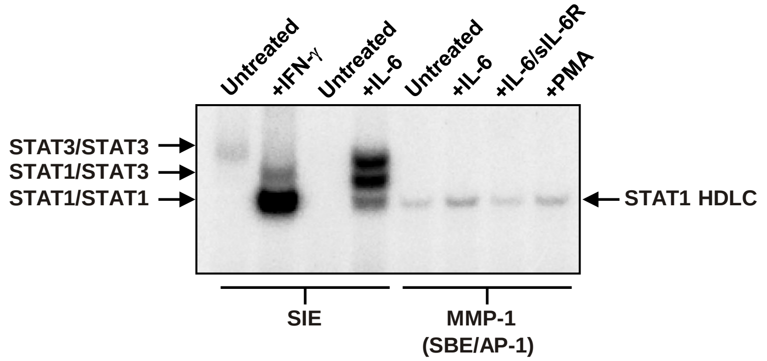
Untreated
+IFN-γ
Untreated
+IL-6
Untreated
+IL-6
+IL-6/sIL-6R
+PMA
STAT3/STAT3
STAT1/STAT3
STAT1/STAT1
STAT1 HDLC
SIE
MMP-1
(SBE/AP-1)

B

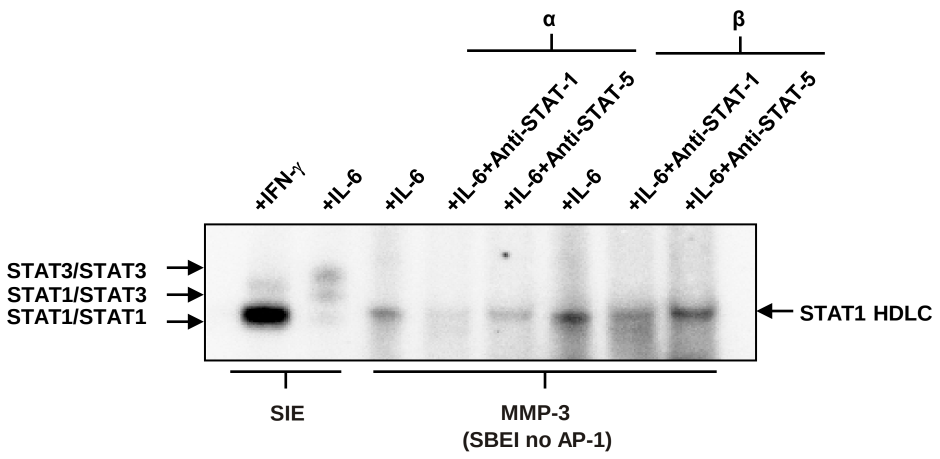
α
β
+IFN-γ
+IL-6
+IL-6
+IL-6+Anti-STAT-1
+IL-6+Anti-STAT-5
+IL-6
+IL-6+Anti-STAT-1
+IL-6+Anti-STAT-5
STAT3/STAT3
STAT1/STAT3
STAT1/STAT1
STAT1 HDLC
SIE
MMP-3
(SBEI no AP-1)

A

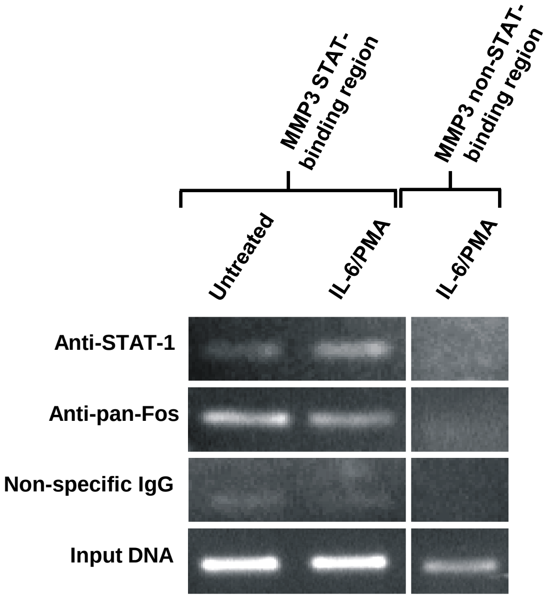
MMP3 STAT-binding region
MMP3 non-STAT-binding region
Untreated
IL-6/PMA
IL-6/PMA
Anti-STAT-1
Anti-pan-Fos
Non-specific IgG
Input DNA

B

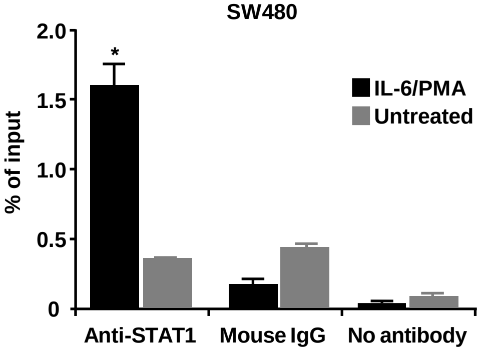
SW480
2.0
1.5
1.0
0.5
0
% of input
*
IL-6/PMA
Untreated
Anti-STAT1
Mouse IgG
No antibody

Cutler *et al*. Fig S6

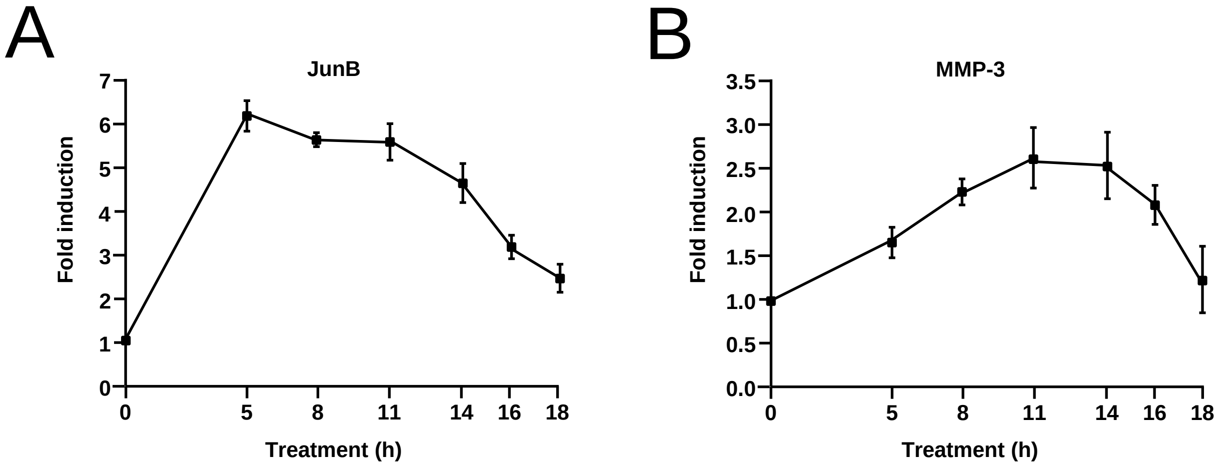

Table S1 Primers used for gene expression analysis.

Gene	Reference	cDNA	Forward (5'-3')	Reverse (5'-3')
MMP-1	NM_002421.2	140bp	ATG TTC AGC TAG CTC AGG ATG A	CCC CGA ATC GTA GTT ATA GCA T
MMP-2	NM_048244.1	110bp	ATG ACG ATG AGC TAT GGA CC	TAC TCC TTG CCA TTG AAC AAG
MMP-3	NM_040737.1	110bp	GGT CAC TTC AGA ACC TTT CCT	ACA GCA GAA TCA ACA GCA TCT T
MMP-7	NM_006273.3	110bp	GAG TGC CAG ATG TTG CAG AAT	GAC ACT AAT CGA TCC ACT GTA A
MMP-9	NM_029934.1	101bp	GGA TAC AGT TTG TTC CTC GTG	GAA GCG GTA CAT AGG GTA CAT
MMP-14	NM_007510.5	58bp	GAG GAA GGA TGG CAA ATT CGT	ACG CCT CAT CAA ACA CCC AAT
KRAS2	NM_004985	112bp	AGT GCC AGT ATT CCC AGA GTT	AGT AAT CAA CTG CAT GCA CCA A
BRAF	NM_004333	102bp	AAT CGG GCT GGT TTC CAA ACA	AGT GGA CAG GAA ACG CAC CA
IRF1	NM_002198	126bp	CAG ATA TCG AGG AGG TGA AAG	TCT CGG CTG GAC TTC GAC TTT
COX2	NM_000963	123bp	GAG TGT GGG ATT TGA CCA GTA T	TGT GTT TGG AGT GGG TTT CAG A
STAT-1	NM_007315	118bp	GGC AAA GAG TGA TCA GAA ACA A	GTT CAG TGA CAT TCA GCA ACT CT
STAT-3	NM_139276	180bp	GCA GAA AGA TAC GAC TGA GG	GCA GAT CAC CCA CAT TCA CT
CMYC	NM_002467	99bp	GCT CCT GGC AAA AGG TCA GA	TGG AGA CGT GGC ACC TCT TGA
SOCS3	NM_003955	102bp	CTT CAG CAT CTC TGT CGG AA	ATC GTA CTG GTC CAG GAA CT
Bcl-2	NM_000633	124bp	AGC ATG CGG CCT CTG TTT GAT	GGC AGG CAT GTT GAC TTC ACT
Bcl-xL	NM_001191	135bp	GTG GAA CTC TAT GGG AAC AAT	GTC TGG TCA TTT CCG ACT GAA
LIF	NM_002309	189bp	AGG TCT TGA AGT GTG CTG TGA A	TTT CCA CTC TGC TCA GCT TCA T
LIFR	NM_002310	260bp	TCC ACC AGA TAC TCC TCA ACA A	CCA GCG GAT TGT GAG CAT TCA
IL-6	NM_000600	240bp	GGG AAC GAA AGA GAA GCT CTA T	TGA GAT GCC GTC GAG GAT GTA
CDH1	NM_004360	235bp	GAC AGA AGA GAG ACT GGG TTA T	GTA TGT GGC AAT GCG TTC TCT A
THBS1	NM_003246	72bp	GGC CAA AGA CGG GTT TCA TTA	ATG GGT CCT GAG TCA GCC AT
CD45	NM_002838	83bp	TGT CTT TGA ACT GAG ACA TTC CA	TGC TCC ACA CTC CAG TTT GTA T
CDK4	NM_000075	114bp	CAC AGT TCG TGA GGT GGC TTT A	TAC CTT GAT CTC CCG GTC AGT T
CTNNB1	NM_001904	231bp	GAA AAT CCA GCG GG ACA AT	TGA GAA AAT CCC TGT TCC CA
*MuMMP-1**	NM_032006	142bp	CTT TGT GCT AAA CCA GGC TGA T	TCC CCT AAA TGT AGT TAT AGC AT
*MuMMP-3**	NM_010809	112bp	ATG TTG GTG GCT TCA GTA CCT	GCA GAA TCC ACA CTC TGT CTT
*MuSTAT-3**	NM_213659	128bp	CGT GGA GCT GTT CAG AAA CTT	GCA ACC TGA CTT TCG TGG TAA
18SrRNA	U13369.1	110bp	CTT AGA GGG ACA AGT GGC G	ACG CTG AGC CAG TCA GTG TA

*Murine

Table S2A STAT binding elements (SBEs).

Gene	Sequence	Relative binding*:		Reference/s
		STAT-1	STAT-3	
murine *C/EBPδ*	TCG **TT** CCCAG **CA** GCA	-	+	[34]
rat *C/EBPβ*	TGT **TT** CCCAG **AA** GTT	+	+	[34]
IRF-1	GAT **TT** CCCCG **AA** TGA	++	+	[35]
c-MYC	GGC **TT**GGCGGG **AA** AAA	+	++	[36]
STAT3	GAG **TT** CCTGG **CA** GTG	-	+	[37]
JunB	CGC **TT** CCTGA **CA** GTG	-	+	[38]
c-*fos*	CAG **TT** CCCGT **CA** ATC	+	++	[35]
ICAM-1	GGT **TT** CCGGG **AA** AGC	++	+	[39, 40, 41]
murine *SAA3*	AAT **TT** CTGGA **AA** TGC	+	+	[42, 43]
murine *Ly6E/A*	TA **TT** CCTGT **AA** GT	++	++	[44]
artificial SBE	**TT** CCGG **AA**	-	+	[43]

*(-) indicates lack of binding, (+) indicates binding and (++) indicates strong binding.

Table S2B Comparison of *MMP* promoter STAT binding element (SBE)-like sequences to consensus SBE sequences.

Consensus GAS	5’ TT(C/A)CNN(G/T)AA 3’
Optimal GAS	5’ TTCGTCGAA 3’
Optimal GAS	5’ TTCCCGTAA 3’
Optimal STAT3-binding element	5’ TTCCGGTAA 3’
MMP-1 SBE	5’ TT***T***CTGGAA 3’*
MMP-3 SBE I	5’ TT***GG***ATGAA 3’
MMP-3 SBE I mutated	5’ TT***GG***AT***C***A***C*** 3’
MMP-3 SBE II	5’ T***GT***TTGGAA 3’#
MMP-3 SBE II mutated	5’ T***GT***TTG***C***A***C*** 3’
MMP-3 SBE III	5’ T***A***C***T***TTGAA 3’
MMP-3 SBE IV	5’ TTCCTGGA***G*** 3’
MMP-3 SBE V	5’ TT***T***CCAGAA 3’

* Non-consensus bases are bold and italicised.
# *mmp*-3 SBE II more closely resembles the consensus sequence in the reverse orientation.

Table S2C Comparison of *MMP* promoter AP-1-binding elements with consensus sequence.

Consensus AP-1-binding element	5’ TGA(C/G)TCA 3’
MMP-1 AP-1-binding element	5’ TGAGTCA 3’
MMP-3 AP-1-binding element	5’ TGAGTCA 3’

Table S3 Oligonucleotides used for electrophoretic mobility shift assays (EMSA).

Name	5' Sequence 3'*
MMP-1 SBE/AP-1 Sense	GATGAGTCAGACAGCCTCTGGCTTTCTGGAAGGG
MMP-1 SBE/AP-1 Anti-sense	CATGCTACTCAGTCTGTCGGAGACCGAAAGACCTTCCCCTAG
MMP-1 SBE Sense	TCGAGACACCTCTGGCTTTCTGGAAGGG
MMP-1 SBE Antisense	CTGTGGAGACCGAAAGACCTTCCCAGCT
MMP-3 SBIV/AP-1 Sense	TCGACTGCCATTTGGATGAAAGCAAGGATGAGTCAAGC
MMP-3 SBEIV/AP-1Antisense	GACGGTAAACCTACTTTCGTTCCTACTCAGTTCGAGCT
MMP-3 SBEI Sense	TCGACTGCCATTTGGATGAAAGCAAG
MMP-3 SBEI Antisense	GACGGTAAACCTACTTTCGTTCAGCT
MMP-3 SBEI Sense	CCGGTACCAAGACAGGAAGCACTTCCTGGAGATTAATC
MMP-3 SBEI Antisense	ATGGTTCTGTCCTTCGTGAAGGACCTCTAATTAGTGAC
MMP-3 SBEII/III Sense	GATCATCCTACTTTGAATTTGGAATGTTTGGAAATGGTC
MMP-3 SBEII/III Antisense	TAGGATGAAACTTAAACCTTACAAACCTTTACCAGTCGA5'
SIE Sense	TCGACGTCATTTCCCGTAAATCCCG
SIE Antisense	GCAGTAAAGGGCATTTAGGGCAGCT

*Shadowed regions indicate location of putative SBE's and AP-1 sites.
